# eHealth for people with multimorbidity: Results from the ICARE4EU project and insights from the “10 e’s” by Gunther Eysenbach

**DOI:** 10.1371/journal.pone.0207292

**Published:** 2018-11-14

**Authors:** Maria Gabriella Melchiorre, Giovanni Lamura, Francesco Barbabella, on behalf of ICARE4EU Consortium

**Affiliations:** Centre for Socio-Economic Research on Ageing, National Institute of Health and Science on Ageing, IRCCS INRCA, Ancona, Italy; Robert Gordon University, UNITED KINGDOM

## Abstract

**Background:**

People with multimorbidity, especially older people, have complex health and social needs, and require an integrated care approach. In this respect, eHealth could be of support. This paper aims to describe the implementation of eHealth technologies in integrated care programs for people with multimorbidity in Europe, and to analyse related benefits and barriers according to outcomes from ICARE4EU study and within the more general conceptual framework of the **“**10 e's” in eHealth by Gunther Eysenbach.

**Methods:**

In 2014, ICARE4EU project identified 101 integrated care programs in 24 European countries. Expert organizations and managers of the programs completed an on-line questionnaire addressing several aspects including the adoption of eHealth. Findings from this questionnaire were analyzed, by linking in particular benefits and barriers of eHealth with the “10 e's” by Eysenbach (Efficiency, Enhancing, Evidence-based, Empowerment, Encouragement, Education, Enabling, Extending, Ethics, and Equity).

**Results:**

Out of 101 programs, 85 adopted eHealth tools, of which 42 focused explicitly on older people. eHealth could improve care integration/management, quality of care/life and cost-efficiency, whereas inadequate funding represents a major barrier. The “10 e's” by Eysenbach seem to show contact points with ICARE4EU findings, in particular when referring to positive aspects of eHealth such as Efficiency and Enhancing quality of care/life, although Empowerment/Education of patients, care Equity and Ethics issues seem crucial in this respect. Encouragement of a new relationship patient-health professional, and Enabling standardized exchange of electronic information, represent further aspects impacting integration/management of care.

**Conclusions:**

Aspects of eHealth, which emerged as benefits and barriers impacting integration/management of care, as well as cost-efficiency and quality of care/life, can be identified on the basis of both ICARE4EU findings and the “10 e's” in eHealth by Eysenbach. They could represent objectives of new policies for supporting the deployment of eHealth technologies within integrated care across Europe.

## Introduction

An increasing number of people in Europe (about 50 million) is suffering from multiple chronic conditions (MCCs) or multimorbidity, in particular 60% of those aged 65 years and over [1]. On one side, multimorbidity implies several and complex health and social needs, high healthcare utilization and the necessity of tailored integrated and patient-centered approaches, On the other side, European health systems are not yet equipped to address the comprehensive care needs of people with multimorbidity [2].

Needs of people with multimorbidity could be met by care services based on innovative technologies, e.g. eHealth tools to support patients’ self-management and multidisciplinary collaboration between professionals [3, 4]. eHealth is the use of Information and Communication Technologies (ICTs) in the healthcare sector. It is defined by the European Commission as “the use of ICTs in health products, services and processes combined with organizational change in healthcare systems and new skills, in order to improve health of citizens, efficiency and productivity in healthcare delivery, and the economic and social value of health” [5]. eHealth tools can play a key role for a better integration of healthcare and social needs. According to the seminal work by Eysenbach [6], eHealth is characterized by being more than a “mere technological development”, that is “a state of mind, a way of thinking, an attitude and commitment for networked, global thinking, to improve healthcare locally, regionally, and worldwide by using information and communication technology”.

Some authors [7] wondered whether consensus had been reached on the definition of eHealth or whether there is a need for a more comprehensive and in-depth review of the literature. In particular, a qualitative study by Pagliari and colleagues [8] found 36 different definitions of eHealth. The original definition by Eysenbach was confirmed, but it was integrated, by adding that eHealth allows a new way of providing traditional healthcare [9].

More recent terms refer to eHealth as “connected health”, that is the integration of technology into healthcare [10], or as “ubiquitous health”, that is the dynamic network of interconnected systems [11]. Furthermore, the expression “intelligent health” is sometimes used to indicate the transformation/analysis of electronic data, obtained by means of eHealth tools, into knowledge and the consequent integration of real-time self-monitoring with assessment of patient’s environment, including also information from family caregivers [12].

The question is that eHealth is a comprehensive and wide concept, an “umbrella” term including various domains, services and applications of ICT on prevention, care, rehabilitation and support, also enabling and interconnecting health service processes and actors, in place and remotely [13]. In order to understand the role of eHealth tools for addressing needs of people with multimorbidity, it seems necessary to group, first of all, eHealth tools within a dedicated framework. According with the classification from the Chronic Care Model (CCM) [14, 15], the key elements of health system/disease management are the following: self-management support, delivery system design, decision support, clinical information systems, and community resources and policies. When the CCM is implemented with the integration of eHealth tools, that can be used to improve the management of chronic illnesses, the model can be re-framed as suggested by the eHealth Enhanced Chronic Care Model (eCCM) [16]. The eCCM in particular showed that eHealth tools can provide important contributions to chronic care, due to their potential impact on: self-management support, e.g. electronic reminders, mobile applications; delivery system design, e.g. tools for supporting care coordination; decision support, e.g. online protocols/guidelines; clinical information systems, e.g. Electronic Health Records (EHRs) including health data of patients regarding prescriptions, medications, vital signs, and laboratory diagnostic examinations. In addition, eHealth education, by means of ICT tools, can provide users with electronic skills when needed, as crucial aspect of self-care.

European countries have implemented some eHealth tools in their healthcare systems, but in most cases they are not integrated in care practices and routines supporting patients with MCCs. In particular, concerning the deployment of eHealth in Europe, Nordic European countries seem to be the leaders in the implementation of eHealth tools, whereas Eastern and Southern Europe include the lesser performing nations, with some exceptions like Spain [13, 17]. A recent World Health Organization (WHO) survey on eHealth [18] showed in particular that 70% of European countries have a national eHealth policy or strategy and 80% have a national legislation to protect the privacy of EHRs, but only 59% have a national EHR system and 69% of these have a legislation concerning its use.

With regard to benefits of using eHealth, previous studies [19–21] showed improved coordination and continuity of care (crucial for older people) by enhanced opportunities for digital data sharing, communication and consultation at distance. Furthermore, valuable reductions in overall hospital admissions, length of stay, and healthcare utilization costs are reported [22]. In particular, the use of eHealth technologies in home care for older people can be cost-effective, even if only family caregivers benefit from it [23]. Patients have the chance to overcome logistic and cost barriers for accessing healthcare services, especially when living in remote and rural communities [24]. Patients can furthermore benefit from improved self-care/management, independent living at home and patient empowerment (especially for the older people), better monitoring and continuity of care, adherence to treatments and maintaining or improving their health status. All this leads to better outcomes for patients [22] and family caregivers [21].

However, there are still various barriers limiting the adoption of eHealth technologies [25–28]. These barriers can be [13]: regulative (e.g. lack of a clear/dedicated legislative framework); technical (e.g. low overall standardization and compatibility/interoperability between different tools, and inadequate technical support and infrastructures); economic (e.g. lack of financing and adequate funding, lack of reimbursement and incentives systems, limited large scale evidence addressing cost-effectiveness of eHealth solutions); and cultural-social (e.g. possible cultural resistance to technology both by professionals and patients, scarce perception of and willingness to use eHealth, and low/lacking integration of the end-users into the development process, particularly for the older people). The lack of adequate eHealth processes also hinders the integration within existing healthcare systems [29, 30].

Benefits and barriers of eHealth are in particular crucial aspects concerning its implementation and adoption process, and Eysenbach in this respect proposed a conceptual framework for framing the potential impact and key factors of eHealth. In his seminal work [6], “10 e's” in eHealth were listed and described: Efficiency, Enhancing, Evidence-based, Empowerment, Encouragement, Education, Enabling, Extending, Ethics, and Equity. As stated by Eysenbach himself, “the ‘e’ in eHealth does not only stand for ‘electronic’ but implies a number of other ‘e's’ which together perhaps best characterize what eHealth is all about or what it should be”.

In the light of these considerations, the aim of this paper is to describe the implementation of eHealth technologies in integrated care programs for multimorbidity in Europe and to analyse in particular their benefits and barriers, within the more general conceptual framework of the **“**10 e's” by Eysenbach. The hypothesis is that, although these were not specifically formulated for eHealth in relation to multimorbidity care and were proposed more than 15 years ago, they may be still valid and have many contact points with ICARE4EU findings, with valuable implications regarding particularly benefits and barriers of eHealth for people with MCCs.

## Materials and methods

### Data sources/Collection

The integrated care programs that are analyzed in this paper come from the European project “Innovating Care for People with Multiple Chronic Conditions in Europe” (ICARE4EU). This project (2013–2016), co-funded by the European Union (EU), mapped innovative care approaches for people with MCCs, which have been developed and implemented in 31 European countries.

Programs were considered for inclusion in the survey when meeting all the following criteria: they targeted adult people (aged 18 and older) with multimorbidity, defined as two or more medically diagnosed chronic or long lasting diseases; they included formalized collaboration(s) between at least two services; they involved one or more medical service(s); they were evaluable or evaluated; they were either still ongoing (in 2014), just finished (less than 24 months before) or about to start (within the following 12 months).

Information on programs was collected with the support of organization experts and program managers in each country included in the study. The experts and managers had expertise on multimorbidity care and were in turn supported by their own extensive network/staff and program leaders. They were asked to identify existing (national, regional and/or local) integrated care programs focusing on multimorbidity in their country, and to report related detailed information by means of a link to a web-survey, and filling in an online questionnaire for each eligible program. The online questionnaire was developed in English and made available in eleven languages. It contained general questions, e.g.: target group/sub-groups of patients, i.e. older people aged 65+, people with physical/cognitive impairments, informal caregivers; specific gender/age as inclusion criteria of the program; particular health problems (e.g. sensory/psychological), as exclusion criteria of the program; main diseases addressed by the program; main objectives, implementation level, types of organizations involved; quality and evaluation of the program. In addition, key elements of multimorbidity care were addressed from the following perspectives: patient-centeredness, e.g. capacity to tailor care according to the specific patient’s needs; management practices and professional competencies, e.g. organizational aspects of providing integrated care; financing mechanisms, e,g, source of funding, savings, incentives; and use of eHealth technologies, e,g, if and how ICT tools were implemented for supporting multimorbidity care. The country experts identified 101 programs, from 24 European countries, responding to the inclusion criteria.

Furthermore, eight good practices (high potential programs) were selected for an in-depth case study analysis, including site visits and further qualitative data collection. To this end, the project team assessed all 101 programs on the basis of a mix of quantitative and qualitative criteria, regarding general dimensions (e.g. evaluation design, perceived sustainability and transferability) and more specific aspects (e.g. level of patient-centeredness, integration of care, use of eHealth technologies and innovativeness in financing mechanisms). This led to identify the ‘top’ eight ‘high potential’ programs, and further information on their contexts and ordinary activities were gathered by means of case studies. The team used a common topic guide-questionnaire for conducting face-to-face and semi-structured in depth interviews with experts/managers and program staff (approximately five interviews per program) in dedicated site visits. Information collected was integrated by additional documents (e.g. internal reports, evaluations) for developing case study reports.

More detailed descriptions of selection of initiative which were visited, in addition to inclusion criteria and data collections, are reported elsewhere [31–33]. For this paper, only information from the quantitative survey was analysed, without including information from the eight good practices.

### Ethics statement

For the ICARE4EU project, no ethical approval was requested, given that the study aimed at collecting secondary data already available to country experts/managers and staff of integrated care programs for people with multimorbidity, without collecting personal/clinical data on sensitive questions regarding patients and family carers. The project team used a web-survey with restricted access (by setting individual access credentials) which was filled in by leading organizations, in addition to some interviews during the site visits to eight ‘high potential’ programs, as explained above. Only general data on the programs was collected. Patients and their family caregivers were not approached. Consequently there were no issues concerning their privacy and anonymity. A written agreement/consent was signed by experts/managers and program staff to contribute to the study and regarding the confidentiality of data collection on care programs selected in their countries.

### Measures

ICARE4EU study distinguished four categories of eHealth tools by their main functions [31, 34] and adopted its own classification by adapting elements of the conceptual frameworks from CCM and eCCM [16]. The four types of eHealth are ICT tools for:

- Remote Consultation, Monitoring and Care: providing remote interaction between patients and health professionals at distance, e.g. consultations/visits by telehealth/telecare, ePrescriptions;
- Self-Management: promoting ability to self-care, used by patients to live more independently, e.g. wearable devices/assistive technologies providing health advice and reminders;
- Healthcare Management: for improving the integration/communication, quality and efficiency of care processes within and between care providers, e.g. EHRs, e-referral systems;
- Health Data Analytics: for analysing data in patient databases and/or clinical evidence for prevention, monitoring and treatment purposes, e.g. Decision Support Systems (DSSs) used by health professionals for clinical decision-making.

Further aspects that were analyzed in the ICARE4EU study were training on use of eHealth, data privacy/security provision, and innovation of the program (as capacity to develop new eHealth tools). Moreover, opinions on potential benefits (improving quality of care, quality of life of patients enrolled, integration/management of care, cost-efficiency) and barriers (inadequate legislative framework, funding, ICT infrastructures, technical-ICT support; lack of skills and cultural resistance among care providers and patients; uncertainty about cost-efficiency; compatibility/interoperability between different eHealth tools; privacy/security issues), were addressed as perceived by experts and program managers. Finally, the provision of incentives for both providers (e.g. for additional staff) and patients (e.g. reimbursement, free access to devices/services), and aspects of evaluation/monitoring of the program, were included. These last two dimensions (incentives, evaluation) in our study were not assessed specifically/only for programs with eHealth adoption (as the other measures mentioned above), but with regard to all the mapped integrated care programs. These were then analyzed only with regard to programs using at least one eHealth tool for the purpose of this paper. More detailed description of measures is reported in a separate paper [31].

The “10 e's” in eHealth by Gunther Eysenbach [6], that were used as conceptual framework to compare ICARE4EU findings, are those already mentioned in the “Introduction” of this paper. They are described with more detail below:

1. *Efficiency*: to increase efficiency in healthcare by decreasing costs;
2. *Enhancing*: the quality of care;
3. *Evidence based*: of eHealth interventions;
4. *Empowerment*: of consumers and patients;
5. *Encouragement*: of a new relationship between patient and health professional;
6. *Education*: of physicians and consumers;
7. *Enabling*: standardized information exchange and communication between providers;
8. *Extending*: the scope of healthcare in a geographical and conceptual sense;
9. *Ethics*: ethical issues, informed consent, privacy;
10. *Equity*: to make healthcare more equitable among the population.

### Data analysis

A quantitative data analysis was performed including the 101 integrated care programs targeting people with multimorbidity on their use of eHealth solutions (e.g. frequencies and bivariate relations). Then the (reported) benefits and barriers of the identified eHealth programs were analysed for further insights. The statistical software SPSS 23.0 was used to carry out the quantitative analyses.

A qualitative data analysis was performed by further exploring findings on programs with eHealth adoption, mainly in terms of benefits and barriers, as well as of EHRs use and access, evaluation of programs, training of patients and providers, incentive mechanisms, and innovation. In this respect, a manual coding process was provided [35]. It led to inductive content analysis [36] of main themes, concepts and relations emerging from the ICARE4EU findings, with the purpose of identifying links with the “10 e's” conceptual framework by Gunther Eysenbach [6]. The aim was therefore to bridge the above mentioned key aspects of eHealth implementation process, as these come from the ICARE4EU findings, with the classification by Eysenbach, and to understand whether these “e’s” were associated to one or more benefits and barriers (and other relevant findings) from our study.

## Results

### Outcomes on eHealth

The findings in this paragraph partly represent a synthesis from a previous publication of the authors, where more detailed description of results is reported [31]. Relevant (and further) data underlying the findings (S1 and S4 Tables), as well as the survey questions used in this study (S1 Text) and the minimal anonymized dataset used for the analyses (S1 Dataset), can be found as Supporting Information in this paper.

Among the 101 integrated care programs mapped on the whole by the ICARE4EU study, 85 included the use at least of one eHealth tool. Out of these 85 programs, 42 focused explicitly on older people aged 65 years and over. The highest number of programs with eHealth tools were identified in Spain (15), followed by Greece, Iceland and Germany (7 in each country), Italy (6) and Finland (5). In the 18 remaining countries, only 1 (e.g. Portugal, Slovenia and Latvia) to 4 programs (e.g. Netherlands, Denmark and Sweden) used at least one eHealth tool. The main reported aim of these programs was to increase multidisciplinary collaboration (85%), whereas organizations and care providers most involved were primary care providers (71%) and General Practitioners (GPs, 80%). The implementation of the initiatives was mostly local and/or regional (78%) and 45% of programs was fully integrated into the regular healthcare services (S1 Table).

Among the eHealth tools which were used, it was reported mostly EHRs (71%), followed by registration databases with patients’ health data for supporting decision-making (64%) and digital communication between care providers (47%). Further eHealth applications (e.g. electronic reminders, computerized DSSs for professionals, and health monitoring and interaction at distance) were not yet widely implemented (S2 Table).

Access to EHRs was mainly allowed to medical care providers involved in care delivery (58%) and less to patients themselves (10%). Moreover, 52% of these programs provided training on the use of eHealth tools to the care providers, but only 24% provided it to the patients and/or family caregivers. About 70% of the surveyed programs assured privacy and confidentiality of health data, and 59% provided data security. Furthermore, the scarce provision of incentives for both providers (28 programs) and patients (only 18 programs) emerged. Concerning the further key issue of evaluation, the results showed that this activity was mainly conducted internally (70%) and less externally (33%). Furthermore, evaluation regarded most the process (69%) and less the outcomes (43%) and cost-effectiveness (30%). Concerning innovation, only 35% of programs specifically developed eHealth tools (24% used existing tools and 15% adapted them) (S3 Table).

The most frequently reported benefits of using eHealth, as perceived by the program managers (Table 1), were on the whole improvements in the management and integration of care (respectively 95% and 93% of program managers agreed) and in the quality of care provided (86%). Other benefits were reported in terms of cost-efficiency of the program (76%) and in the quality of life of patients enrolled (70%). All these benefits were moreover more evident with regard to the integrated programs targeting the older people (Table A in S4 Table)

**Table 1:** Benefits of using eHealth tools included in the programs from the ICARE4EU study (% of agree)^a^.

Benefits	Programs with at least 1 eHealth tool, N = 59	
	N	%
Management of care	56	95
Integration of care	55	93
Quality of care	51	86
Cost-efficiency	45	76
Quality of life	41	70

^**a**^ Multiple answers were allowed.

Inadequate funding (60%) emerged as the main barrier hampering the use of eHealth tools in integrated care programs (Table 2). Inadequate interoperability and technical infrastructure, lack of skills among patients/providers, and lack of a legislative framework also emerged (45–50%). In addition, uncertainty on cost-efficiency of the program, privacy and security issues, resistance to adopt eHealth tools by providers (33%) and patients (22%) were mentioned with a lower rate of agree (22–40%). Regarding barriers, large differences between programs targeting adults or older people were not found (Table B in S4 Table). In particular, the lack of skills among patients seems at the same level in programs for both groups.

**Table 2:** Barriers for using eHealth tools included in the programs from the ICARE4EU study (% of agree) ^a^.

Barriers	Programs with at least 1 eHealth tool, N = 58	
	N	%
Inadequate funding	35	60
Compatibility between different eHealth tools	32	55
Inadequate technical ICT support	32	55
Inadequate ICT infrastructures	31	53
Lack of skills among patients	30	52
Inadequate legislative framework	29	50
Lack of skills among providers	26	45
Uncertainty of cost-efficiency	23	40
Privacy/security issues	20	35
Resistance by care providers	19	33
Cultural resistance	15	26
Resistance by patients	13	22

^**a**^ Multiple answers were allowed.

### Outcomes on eHealth and the “10 e’s” by Eysenbach

An analytic framework (Table 3) was developed in order to link our findings to the conceptual scheme of the “10 e's” in eHealth by Gunther Eysenbach [6]. Benefits and barriers were mainly considered, in addition to other selected results (last column of Table 3, e.g. EHRs use and access, evaluation of programs, training of patients/providers, incentives mechanism and innovation) which were considered useful, as further potential barriers or consequences of other barriers, to reinforce our considerations.

**Table 3:** Benefits and barriers of/for using eHealth tools: ICARE4EU findings and framework by Eysenbach.

“10 e's” in eHealth by	eHealth Benefits from	eHealth Barriers from	Further findings from
Gunther Eysenbach	ICARE4EU study	ICARE4EU study	ICARE4EU study
*Efficiency*	Cost-efficiency	Inadequate funding	Scarce provision of incentives
*Enhancing quality*	Quality of care	Uncertainty about cost-	Low both internal/external
*Evidence based*	Quality of life	efficiency	evaluation
			Low innovation (eHealth tool
			developed ad hoc for the
			program)
*Empowerment*	Quality of care	Lack of skill of patients	Mainly training to care providers
*Ethics*	Quality of life	and care providers	and less to patients
*Education*		Resistance by patients	Low access to EHRs by patients
*Equity*		and care providers	
		Cultural resistance	
		Inadequate funding	
		Inadequate legislative	
		framework	
		Privacy/security issues	
*Encouragement*	Integration of care	Resistance by patients	Low access to EHRs by patients
*Enabling*	Management of care	and care providers	Scarce provision of incentives
*Extending*		Cultural resistance	Low innovation (eHealth tool
		Inadequate funding	developed ad hoc for the
		Compatibility between different	program)
		eHealth tools	
		Inadequate technical support	
		Inadequate ICT infrastructures	
		Privacy/security issues	

ICARE4EU findings, in particular on benefits and barriers, were analysed without their respective percentages and without listing them in order of importance as perceived/referred by the program managers. Conversely, all of them were explored as crucial aspects of eHealth adoption, to better compare our results with the specific “10 e's” by Eysenbach. In particular, in many cases more results could be associated to these essential “e’s”.

Both Eysenbach and ICARE4EU study put in evidence (cost-) *Efficiency* and *Enhancing quality* of care and life. These aspects represent potential benefits of eHealth which could be hampered by inadequate funding and incentives, and by the lack of appropriate program evaluations, which conversely could provide useful *Evidence based* data on efficiency itself. Lack of adequate financing mechanism and uncertainty about cost-efficiency in turn could affect the possibility of innovation, that is developing new ad hoc eHealth tools.

Furthermore, *Empowerment* of patients, *Ethics* issues, *Education* of patients/health professionals, and care *Equity*, are further aspects of eHealth related to quality of care/life. In this respect, other aspects could represent crucial obstacles for the adoption of eHealth solutions, such as lack of skills of patients and providers, their cultural resistance “to change”, inadequate legislative framework and funding, and privacy/security issues, in addition to few training opportunities for patients. Limited funding can further negatively impact investment in education and training services for both users and providers. This negative context then could in turn impact empowerment and lead to low access to EHRs by patients themselves.

Finally, other aspects represent conditions that could positively impact and improve both integration and management of care due to eHealth adoption, like: *Encouragement* of a new relationship between patient and health professional, *Enabling* standardized information exchange between providers, and *Extending* the scope of healthcare in a geographical and conceptual sense. Also these potential benefits can find obstacles, such as general cultural resistance to adopt eHealth, inadequate funding and incentives, lack of technical infrastructure and support, problems of compatibility and interoperability between different tools, and privacy/security issues. These aspects moreover could affect the possibility to “encourage” access to EHRs by patients, and the possibility of innovation with eHealth tools.

## Discussion

Drawing on evidence from our ICARE4EU study, the use of eHealth tools, as referred by country experts and program managers, seemed to show some potential benefits, mainly as support for management/integration of care, as well cost-efficiency and quality of care and life. On the other side, findings suggested some issues and challenges, which could represent strong barriers (infrastructural, technical, economic, legislative and practical) to the wider deployment of eHealth, with a consequent negative impact on the quality of care and quality of life of patients. In particular, benefits were more evident with regard to programs focusing older people, whereas barriers did not show substantial differences between programs targeting adults or older people.

Overall, these interrelated issues were highlighted (in similar terms) also by Eysenbach, although his work was not exclusively focused on eHealth solutions targeting people with multimorbidity. In this respect, some further considerations and reflections can be highlighted on the basis of both ICARE4EU findings and the “10 e's” by Eysenbach [6], with large support from existing literature, in order to identify ways of “bringing about change” in the general implementation process of eHealth [37].

### Efficiency, Enhancing quality of care, Evidence based initiatives

*Efficiency*, *Enhancing quality* of care, and *Evidence based* programs represent aspects of eHealth which are strictly related each other, given that increasing efficiency could involve both reducing costs and improving quality of care and life, in particular for the older people, (as emerged from ICARE4EU data), but in the same time eHealth interventions should be evidence-based, that is with expected effectiveness and efficiency supported by scientific data and facts produced by rigorous evaluations.

Increasing *Efficiency* in healthcare is one potential key”promise” of eHealth, and cost-savings could result from avoided hospitalizations, or “duplicative/unnecessary diagnostic/therapeutic interventions, through enhanced communication possibilities between health care establishments, and through patient involvement” [6]. de Bruin and colleagues [38] also focused on improvements to existing integrated care initiatives (in particular for older people with multiple health and social care needs), especially in relation to efficiency, defined as effective use of infrastructure, resources, equipment and technology for sustainability and reduction in healthcare spending. Such a positive economic impact of eHealth, as reduction in hospital days per patient and overall cost-savings (as direct consequence of better clinical outcomes and well-being of the patients), is supported for instance by results of introducing home telemonitoring in various countries (e.g. Netherlands, UK and Germany). Furthermore, savings from adoption of digital prescriptions are reported [39]. In particular, the increased multi-professional collaboration, thanks to innovative technologies, can lead to savings [40].

These circumstances can have positive consequences for *Enhancing quality* of care and life [41], by “allowing comparisons between different providers”, in order to choose the best opportunities in terms of quality [6]. European Commission [42] stressed the importance to direct national eHealth governance towards delivery of citizen-centric healthcare, with patients actively involved for the maintenance of their own health. With particular regard to older persons, this new vision of care produces social returns, as their improved independence at home (e.g. by reducing falls, preventing and combating depression and isolation, and developing informal networks), in addition to improved quality of life of their family caregivers [43]. In this respect, a new frontier is represented by eHealth platform for older people and their caregivers, providing information and support to facilitate and optimize caregivers’ work and to improve elders’ quality of life [44, 45]. Recent findings confirmed that the use of the network consulting room (e.g. mobile platform, with patient interacting through video, voice and text with the doctors) can improve the quality of life of patients and reduce the number of re-hospitalization [46]. A recent literature review suggested that web-based interventions, for supporting informal caregivers of adult people with chronic conditions living in the community, can improve general health outcomes of caregivers themselves (e.g. reduced depressive and anxiety symptoms) [47, 48].

The possibility to have economic support for clinicians seems to play a role when adopting technological innovations [49]. In general, the lack of adequate financing sources, as well as limited incentives and reimbursement mechanisms (as emerged from ICARE4EU results), have a negative impact on a widespread utilization of eHealth applications, and in particular of telehealth services [50]. As with other technological innovations, some clinicians will adopt them readily, whereas others will need incentives and support. Conversely, it seems strategic to develop innovative and sustainable financing and reimbursement mechanisms for eHealth, and to assess how financial flows in healthcare and welfare systems may provide incentives for telehealth provision [25, 51]. Incentives should regard patients and health workforce, and should involve industry and other relevant stakeholders for a full success in eHealth adoption [52]. Furthermore, in the light of a multidimensional approach, adequate financial schemes should try to overcome the separation of budget for health and social care services, currently existing in many European countries [26]. In this respect, the use of “joint budgets” across health and social care sectors could represents a good policy option to support the use of eHealth and to promote continuity of care for people with MCCs [53]. In particular, shared “service centres” could be of help in facilitating the wider deployment of telemedicine [54].

The fact that, according with ICARE4EU findings, inadequate funding represents a major barrier hindering the adoption of health technologies, suggests that the financial context in some countries (for instance in Eastern European countries) may affect the development of reforms directed at the care for people with multimorbidity [55], including the exploitation of eHealth potential. Moreover, the scarce provision of incentives for both providers (e.g. for additional staff) and patients (e.g. increased reimbursement, free access to devices/services), makes difficult a wider adoption of integrated programs using eHealth tools, especially in the light of the financial constrains in public healthcare budgets for most European countries [56]. In particular, recent findings highlighted that, although policies on remote monitoring existed in some European countries together with pilot projects, the need for capital investment was not satisfied and formal incentives were scarce [57].

Another aspect impacting *Efficiency and Enhancing quality* of care and life is the lack of systematic evaluations of programs adopting eHealth, either conducted internally or externally. When available, evaluation seems mainly internal in most cases, according to ICARE4EU findings. Evaluation could provide useful *Evidence based* data on cost-efficiency itself. eHealth interventions should be evidence-based, and effectiveness and efficiency “should not be assumed” but supported by rigorous scientific evaluation [6]. Currently, there is a lack in the number of large rigorous clinical trials and field research studies, which could provide evidence on health outcomes and other effects [41, 50, 58]. Conversely, it seems crucial to conduct, synthesise and use evidence from large-scale studies on (cost-) effectiveness of eHealth applications (e.g. on satisfaction of service users and health/social care professionals, and related costs) [26].

Large-scale interventions are especially needed to evaluate the impact of eHealth tools, rather than small-scale research, since these latter ones cannot evaluate effectively the impact itself [34]. “The impact of eHealth technologies is sometimes questioned because of a mismatch between the postulated benefits and actual outcomes” [59]. Moreover, it should be highlighted that changes involving eHealth adoption are challenges which require sufficient financial resources and additional investments for a long period of time, in order to have eHealth services actually “paying off” [26]. As a consequence of this, there is a need for long-term studies to verify the sustainability of benefits eventually emerging from short-term trials [60]. In order to have eHealth technologies confirming their durability and acceptance for patients on the long-term [58], care programs with eHealth should be designed with “evaluation in mind” and with considerations on possible integration within the healthcare system, especially in relation to benefits and effectiveness within routine settings [61]. In particular, the economic impact of eHealth seems urgent to be evaluated with regard to older people with MCCs, given that studies referred an exponential association between multimorbidity in later life and healthcare costs [62], in order to adapt care programs at best to their specific needs [63].

The lack of sufficient empirical evidence on costs and benefits, thus generating uncertainty about cost-efficiency, make healthcare professionals often “skeptical” on the potential of eHealth technologies. It seems important to monitor outcomes in order to better inform and drive decisions of relevant stakeholders (e.g. on human resources and financing requirements). Conversely, findings from the ICARE4EU study showed that evaluation of programs regarded most the process and less the outcomes. Motivated stakeholders could be available to use e.g. telehealth solutions, but evidence is needed in order to invest in digital health services [64]. Systematic evaluation of eHealth interventions and scientific evidence could convince policymakers, who often refer low access to good quality evidence, and the lack of timely research output, as crucial barriers to the use of evidence [65]. A recent review [66] in particular highlighted the importance to improve eHealth evaluations by measuring adherence of outcomes to the “intended use” of different eHealth technologies.

The lack of adequate financing and incentives mechanisms in turn negatively impacts the possibility to provide appropriate internal and external evaluations of the eHealth tools. This may limit the possibility of innovation and the development of new eHealth tools, given that investors need to have robust evidence on economic returns, in order to be willing to finance new care programs with eHealth [59, 67]. In this regard, from ICARE4EU results emerged that only few programs specifically developed new eHealth tools. These programs should show their sustainability depending on their evidence based ability to generate social and economic returns (e.g. by generating savings), with a business model that is affordable for the users [43]. Digital transformation is a great opportunity to increase health care performance “by lowering cost and improving quality of care”, but in this respect, and regarding an economic scale, “business models can be strengthened” [68].

### Empowerment, Ethics, Education, Equity

*Empowerment* of patients, *Ethics* issues, *Education* of patients and health professionals, in addition to care *Equity*, are further aspects of eHealth impacting quality of care and quality of life, thus enabling patient-centered care. In this respect, there are some obstacles preventing a fruitful adoption of eHealth solutions, as emerged also from the ICARE4EU study, such as the lack of guarantees of privacy and confidentiality of data, inadequate legislative framework, lack of skills of patient/providers, their cultural resistance “to change”, and few training opportunities for patients.

*Empowerment* of patients, in particular, implies to support self-management of people with multimorbidity living at home, through tools providing feedback or check of adherence to treatment, including tools that educate and empower them in self-care [34, 69]. eHealth is indicated as a key driver for developing patients’ empowerment [27, 70]. *Empowerment* of patients, and their involvement in decision making processes, can be reached, for instance, “by making personal electronic records accessible to consumers over the Internet” [6]. The opportunity to access these records (EHRs in ICARE4EU study), makes individuals more active and controllers and responsible of their own health data concerning disease, treatment and prevention, with the right to make decisions on management of their wellbeing, and to be informed about how their health data will be used. The possibility for patients to access them can increase their trust in care providers, and this can allow easier communication with health professionals [71]. In this respect more than eight out of ten Europeans [72] indicate that they do not feel that they have a complete control of their personal data online. ICARE4EU study also highlighted that access to EHRs was allowed to patients only in few cases, and that in particular a lack of digital skills among patients and providers represented a barrier in about half of the mapped care programs adopting eHealth tools for multimorbidity. In addition, the general cultural resistance to understand and appreciate the usefulness of eHealth tools, and few dedicated opportunities of education and training on eHealth, especially for patients, emerged as aspects to be considered.

*Empowerment* of patients and online self-management involve in particular *Ethics* aspects concerning privacy/security and informed consent by patients [6], and this in turn could involve aspects related to training needs for patients on these issues. In particular training for users of telecare is an important factor for improving patient safety [73]. These aspects should be mandatory when adopting eHealth, within a clear and dedicated legislative framework. In particular, potential opportunities and threats of eHealth should be identified before designing/planning an ethical framework [74]. ICARE4EU findings indicated that not all the surveyed programs assured privacy and security of personal medical data. Moreover, WHO [18] highlighted that currently not all European countries have fully addressed this issue. As already put in evidence, 80% have a national legislation protecting the privacy of EHRs, but only 59% have somehow a national EHR system, and 69% have a legislation concerning its use. The lack of legal and regulatory issues should be addressed, in addition to privacy and security issues, especially when patients are moving from an institutional setting to their homes (protected discharge) [26]. Moreover, the implementation of guidelines assuring a safe use of digital health tools and data could be of great help [75].

*Education* of “consumers” and “of physicians through online sources (continuing medical education)” [6], in addition to education of formal and informal caregivers, represent key aspects impacting the *Empowerment* issue. eHealth enables efficiency, quality and continuity of care but requires adequate education of all actors involved, on potential capabilities and benefits coming from ICTs. Health professionals, in particular, could have great help from online systems, e.g. eLearning platforms for vocational training [76]. However, first of all there is a need of more digital skills training and support in order to have competencies in clinical informatics for medical education [19, 77].

eLearning, in particular as computer-based educational intervention for GPs, seems effective in enhancing their competencies in communication with older patients [78]. With regard to patients, it is important to enhance. “eHealth literacy” or ‘digital health literacy’ as a key pre-condition for the acceptance of eHealth tools and their use for self-care and management [16]. Having ‘digital health literacy’ means to have adequate skills in order to access, understand, use and benefit from both electronic health information and tools [79]. More in general, older patients have a low ‘health literacy’, intended as the capacity to comprehend basic health information from healthcare providers or from traditional sources (e.g. instructions for medicines), and it is more likely that they have also a low ‘digital health literacy’, with the consequent need of particular assistance in using both traditional and ICT-based information, care and self-management [29, 80]. In most cases, there is indeed a digital divide in older adults, due to their decline in cognitive and physical functionalities related to the aging process, and to their negative attitudes toward technologies [81, 82]. The digital divide between young and older people is also put in evidence by Eysenbach [6]. ICARE4EU data report that the lack of skills (for using eHealth tools) among patients seems at the same level with regard to care programs for both adult people in general and older people in specific. Moreover, when implementing new eHealth applications, professional care staff can play a key role, but professionals should themselves first of all believe in the potential benefits of new technologies for the patients [83]. Nursing personnel represents in particular a large group of health care professionals from whom a successful implementation of eHealth applications depends. They could be sustained by the support of adequate training enhancing the adoption of eHealth by patients [70]. Nurse practitioners educated/empowered in/with telehealth could strongly support health care, and related innovations, within practice [84].

Limited funding can further negatively impact investment in education and training services, for both users and providers, on eHealth tools use. This negative context then could further lead to unequal access to technology among people. *Equity* and social inclusion in eHealth adoption represent thus other issues to be considered. “To make health care more equitable is one of the promises of e-health”, but policy actions seem necessary to ensure “equitable access for all” [6]. Health interventions delivered via internet or mobile phone apps, can provide “promising alternative health-care delivery models”, especially for marginalized and excluded populations [85]. Inequalities in eHealth adoption could lead, for instance, to low access to EHRs by some group of more disadvantaged/vulnerable patients who are socially isolated, e.g. without digital skills, with lower socioeconomic status, living in deprived and rural areas, with lacking technological infrastructure, and with low mobility [86]. The digital divide is not the only one existing between young and older people, but it is also represented by the gap between e.g. rural and urban populations, and rich and poor social groups [6]. Furthermore, the presence of disabilities may exacerbate the digital divide [87]. Especially in rural and socially deprived areas, with low (or no) availability of healthcare services, eHealth tools can lead to better *Equity* in accessing healthcare, e.g. by enabling remote consultations, treatment and rehabilitation [34].

Inequity in access to health and social care, as well as access to and use of eHealth solutions, are crucial aspects regarding in particular the care for people with multimorbidity [80]. Although eHealth brings the promise to reduce social health inequalities, it could increase them, if the designers do not keep in consideration that an eHealth application could be effective for one group, and with negative consequences for another one, based on physical, cognitive, or cultural differences. In particular, eHealth tools could increase social disadvantages for older people or those with low income. These aspects need to be addressed in order to reach a universal access to eHealth [88]. Moreover, how different eHealth technologies are accessed and used, and can “reduce or (re)produce” social inequalities in health, depend on the context in which institutional and political context they work [89].

The low impact of eHealth technologies on equity for healthcare access is also due to the fact that users are often only marginally involved in the development [59]. This lacking patient-centeredness in turns could produce usability obstacles [90], or high attrition rates, that is the proportion of consumers who stop using technologies which are not perceived as useful or easy-to-use [91]. Conversely, the development of “need-driven” eHealth tools prototype, by involving especially older people end users, could be more effective [92]. Several study findings in particular suggest that patient portals should allow easy visually engaging and user-friendly navigation, to be realized by an early-stage involvement of patients in design and development of eHealth solutions [93–95].

### Encouragement, Enabling, Extending

*Encouragement* of a new interaction between patient and health staff, *Enabling* communication in healthcare context, and *Extending* the scope of healthcare in a geographical and conceptual meaning, these all represent factors that could positively impact integration and management of care. In particular, integration and management of care were referred/perceived as key potential benefits of eHealth by program managers interviewed in the ICARE4EU study, especially for older people. On the other side, an appropriate management (and coordination) of the eHealth tools (e.g. objectives, organisation), respective responsibilities of actors and allocated resources is needed, given that it impacts the outcomes from eHealth technologies themselves [59].

*Encouragement* **“**towards a true partnership, where decisions are made in a shared manner” [6] could in particular facilitate a “multidimensional approach towards professional change management” [26]. Such an approach engages all involved actors including patients, and regards changing of organisation, work processes and behaviours, which all represent difficult goals to be achieved when improving health and social care service delivery. eHealth applications can contribute to renew the patient-professional relationship, with impact on the empowerment of patient [27] at the micro level of care, i.e. regarding strictly the relation patient-physician, and at the meso/macro level of care, i.e. on institutional and policy levels [96]. A recent study [97] put in evidence that eHealth might enhance interactions with patients and their effective care, and that new technologies may be of help in managing changing demands of patients themselves. eHealth indeed develops home-based solutions which are integrated within the national/regional healthcare systems, with improvement of functionalities such as information, education, and communication of/with patient [74, 98]. The communication and exchange of electronic information between patients and providers should especially regards the relationship with the GP and primary care providers, who are the key actors caring for older person with multimorbidity [3]. In particular, GP is perceived by family carers as a real “support service” in terms of information, counselling and emotional/psychological support [99]. According to data from the Survey of Health, Ageing and Retirement in Europe (SHARE), regarding people aged 50 years and over in 16 European Countries in 2011–2012, multimorbidity is linked to increased primary care utilization, and particularly to increased number of visits by GP [100]. GPs have a crucial role regarding health literacy of older patients [101], given their long-standing confidence and familiarity with them [102]. However, some authors [103] found that GPs used eHealth tools more frequently for their own needs and less frequently for their patients.

Beside a new relation patient-physician, *Enabling* “information exchange and communication in a standardized way between health care establishments” [6] represents a further aim for eHealth tools. Their adoption within the healthcare system implies a full and standardized coordination of the communications among healthcare professionals, patients and informal caregivers, especially when disease management regards multimorbidity, with several professionals involved [59, 104]. Currently eHealth is not yet a major component in most healthcare systems, and standardization should be increased in both national and European contexts in order to achieve its potential [34]. Including all relevant stakeholders in such a process could add success to the final desired outcomes [30].

The role of technology in facilitating the integration, communication and sharing of information among providers/professionals and between professionals/providers and patients, “wherever they are based”, seems a crucial/strategic issue [105]. *Extending* the aim of healthcare “in both a geographical sense as well as in a conceptual sense” means in particular that eHealth services, ranging from “simple advice to more complex interventions” can be obtained from online global providers [6]. Via eHealth patients can reach various providers which are located in different countries, in order to have for instance a first or second opinion on a specific treatment, or to use a specific online healthcare service, especially useful for those living in remote and “conventionally” inaccessible areas [106]. Telehealth in particular can overcome social and geographic inequalities, by allowing more people to receive health care [50]. The possibility to connect suppliers and users in the whole Europe, allowed by communication technologies through an integrated/coordinated involvement of professionals and stakeholders, seems essential in the cross-border healthcare services, thus improving the continuity and quality of care across Europe [107].

The potential of eHealth in terms of *Encouragement* of a new interaction patient-health professionals, *Enabling* standardized communications, and *Extending* the scope of healthcare, as described above, can face obstacles as those indicated also by ICARE4EU results, e.g. the cultural resistance to adopt eHealth (both by patients and providers), that in turn could affect the possibility to “encourage” the access to healthcare services enabled by eHealth technologies. It is to keep in consideration that a good patient-physician relationship could be hard to reach, given that for the patient it is difficult to trust online rather than face-to-face, and moreover sometimes with unknown and different professionals, with whom the patient has not yet a relationship. This in turn could lead to fragmented and inappropriate healthcare [50]. In this respect, a “careful management” of patient-physician relationship could conversely encourage the adoption of health technology, to support especially people with chronic disease [108]. Video consultations in particular seem to work better than face-to-face consultations when health professionals and patients already know and trust each other [49]. Some authors in a recent study [109] highlighted however that the quality of patient-provider communication did not differ significantly between web-based and face-to-face consultations, and both seem to offer the same satisfaction/interaction level.

For *Enabling* communication in a standardized form between providers/professionals, and for *Extending* the scope of healthcare in a geographical and conceptual meaning, a good eHealth governance also seems particularly crucial, e.g. the provision of adequate funding and incentives, as well as technical, institutional/organisational structures [110]. Especially telehealth services has potential to reach successful outcomes, but its applicability could remain low due to technology and infrastructure required, and related costs [111]. These barriers in turn could affect the possibility of innovation, as development of new eHealth tools. In a general sense, willingness to innovate by providers and stakeholders should be fostered with opportunities raising dialogue, exchange of standardized information and awareness on potential benefits of eHealth [26]. The involvement of providers and stakeholders in productive discussions and decision-making process about possible healthcare innovation could facilitate the acceptance of new ICT-based tools [112]. Moreover, an effective innovation could be achieved only by supporting interoperability and compatibility of technology between various and different healthcare ICT applications and systems [113]. This represents a challenge to be carefully managed [114], both within and between European countries, in order to implement a homogenous/harmonized framework for the exchange of health information used in cross-border services [30, 107]. In particular, EHRs, which are based on data reported by healthcare providers, are often stored in a fragmented way in different structures. These aspects impact the interoperability and the related data exchange between health professionals and providers [115]. Both technical and semantic interoperability of different eHealth applications should then be guaranteed, in order to obtain benefits from integrated healthcare information systems [26].

Furthermore, *Enabling* a standardized communication faces in particular the barrier of privacy and security issues. Collaboration between care professionals, when supported by adoption of eHealth options, remains difficult in practice, partly due to a lacking or not clear legislation for the protection of privacy and security [80], as already stated above regarding particularly *Ethics* aspects of eHealth. When healthcare organizations substitute traditional care with “care at distance”, it is very important that secure systems are used [116]. To assure private and secure communication could encourage patients to adopt a new online relation with health professionals, in particular with regard to telehealth use. In this case, several types of patient safety risks emerged, mainly related to various tasks and practices and to the personal characteristics and capabilities of users/informal caregivers [73].

### Limitations

The ICARE4EU study presents some limitations [31–33]. First, our overview of integrated care programs for multimorbidity in European countries reported the impact of eHealth applications as perceived by country-experts and program managers, without including the impact of eHealth on quality of life and quality of care as perceived by patients and their caregivers, or the impact on integration of care as perceived by care providers. Second, the survey was dependent on the personal expertise of country-experts and program managers participating in the survey. In some cases, they might not have had complete knowledge of all multimorbidity care approaches and programs operating in their countries. Third, eHealth aspects that were considered relevant for multimorbidity care were mapped, but comprehensiveness of data collection on the phenomenon cannot be assured. Moreover, our analysis has some limitations: the “10 e's” in eHealth by Gunther Eysenbach are not specific for technologies adopted within integrated care programs for multimorbidity, whereas it was conversely the aim of our study, and moreover they were identified in 2001; in many cases further benefits/barriers/other results could be associated to each of these essential “e’s”, but it was decided to highlight those most supported by ICARE4EU findings and previous/current literature.

Despite these limitations, regarding both the ICARE4EU study and the analysis that was carried out for the purpose of this paper, the relatively high number of eHealth initiatives which were mapped in the context of multimorbidity care, contributed to raise knowledge in the field, and confirmed the relevance of the “e’s” in eHealth focused by Eysenbach more than 15 years ago. These seem crucial factors still valid and applicable in the current context of eHealth deployment for multimorbidity care in Europe.

## Conclusions

The increasing incidence of chronic diseases, and the issue of how to appropriately meet the complex care needs of people, especially those with multimorbidity and mainly elderly, calls into the question the role of eHealth options within healthcare services. In this respect, some important aspects impacting care integration and management, as well as cost-efficiency and quality of care and life, can be identified on the basis of both ICARE4EU findings and the “10 e's” in eHealth by Gunther Eysenbach [6]. These aspects could be considered as potential objectives of new policies which could support the development and use of eHealth technologies within integrated care across Europe [34].

First of all, for a positive adoption of eHealth tools the following aspects seem crucial: developing adequate/clear legal frameworks (e.g. on access to EHRs by patients), with attention to ethics aspects such as privacy/security issues; to provide innovative and sustainable funding systems, incentives and reimbursement mechanisms for large scale implementation of eHealth; to have adequate technical, institutional and organisational infrastructures facilitating communications between care providers; to assure interoperability and compatibility of technologies between different ICT tools/systems, and standardization of processes. A whole “digital framework”, potentially at a national/regional level, seems thus needed, in order to understand the complex interactions between the different eHealth tools [58].

Moreover, a cultural acceptance “to change” should be based on the provision of education and training to patients, family caregivers and health professionals on digital health literacy, which contribute in a complementary way to achieve patient-centred care, empowerment and self-management. In this respect, it seems important to ensure equitable access to eHealth applications to more vulnerable subgroups of the population with scarce digital skills, with particular attention to older persons, who could greatly benefit from eHealth adoption.

However, to have successful outcomes of eHealth, thus enhancing the quality of care and life, it seems crucial to carry out large-scale and longitudinal research studies producing robust evidence. Internal and external evaluation are required for verifying the impact of eHealth tools on patients and caregivers, on cost-effectiveness and efficiency of eHealth applications, and on usability and appropriateness of such new technologies. Innovation and development of new eHealth tools could further be based on evidence. In particular, Eysenbach stated that, in addition to the essential “10 e's” in eHealth, eHealths itself “should definitely exist!”. This could mean that it is fundamental to create eHealth tools, to innovate and provide new technologies, e.g. specifically developed for a particular care program, when possible, instead of using or adapting existing tools.

A holistic and inclusive approach seems needed to address successfully issues such as technology, management and finance, in addition to human/contextual factors and stakeholders’ involvement, when planning, implementing, and evaluating eHealth applications [59]. Such an approach could really extend the scope of healthcare in a geographical and conceptual sense, and promote new opportunities for collaboration and investments in relevant eHealth technologies. In particular, in order to increase the possibility of success, future research on eHealth interventions should be directed towards the impact in the quality of care, with attention to management and patient-centered care [117]. The recent Communication from the European Commission [118] highlights particularly personalized medicine as a priority of eHealth, besides citizen empowerment and secure/safe access to electronic data. Researchers, practitioners and policy makers should finally aim to work together for achieving the final promise of eHealth tools for patients and most disadvantaged social groups.

## Supporting information

S1 Table Number of programs using at least one eHealth tool by main general aspects. (DOCX)

S2 Table Number of programs using at least one eHealth tool by categories. (DOCX)

S3 Table Number of programs using at least one eHealth tool by some specific aspects. (DOCX)

S4 Table Programs using at least one eHealth tool and focusing older people 65+. Benefits and barriers (number of agree). (DOCX)

S1 Text Survey questions used in the study. (DOCX)

S1 Dataset Minimal dataset used for the analyses. (ZIP)
