# Supplementary material for: eHealth for people with multimorbidity: Results from the ICARE4EU project and insights from the “10 e’s” by Gunther Eysenbach
Source: PLoS One. 2018 Nov 14;13(11):e0207292. doi: 10.1371/journal.pone.0207292 (PMC6241125; doi:10.1371/journal.pone.0207292)
Supplement: S1 Table — (DOCX) [file pone.0207292.s001.docx]

**S1: Number of programs using at least one eHealth tool by main general aspects**

|  | All Programs N=85 |
| --- | --- |
| **Main objectives^a^** |  |
| Increasing multidisciplinary collaboration | 72 |
| Improving patient involvement | 64 |
| Improving care coordination | 61 |
| Reducing hospital admissions | 60 |
| **Organizations involved^a^** |  |
| Primary care | 60 |
| General hospital | 53 |
| University hospital | 36 |
| **Care providers involved^a^** |  |
| General Practitioner (GP) | 68 |
| Medical specialists | 59 |
| Districts/community nurses | 47 |
| Hospital/specialized nurses | 47 |
| **Types of care and support provided by programs^a^** |  |
| Medical care | 67 |
| Prevention/delay of deterioration | 58 |
| Nursing care | 56 |
| Lifestyle and health behaviour | 56 |
| **Integration level** |  |
| Small scale (pilot) program | 22 |
| Well-established and comprehensive program | 25 |
| Fully integrated in the regular healthcare system | 38 |
| **Implementation level** |  |
| Local | 22 |
| Regional | 29 |
| Local/regional, as part of a national program | 15 |
| National | 11 |
| National, as part of international programs | 5 |
| Inter-/Supra-national | 3 |

^a^ Multiple answers were allowed.
