# Supplementary material for: eHealth for people with multimorbidity: Results from the ICARE4EU project and insights from the “10 e’s” by Gunther Eysenbach
Source: PLoS One. 2018 Nov 14;13(11):e0207292. doi: 10.1371/journal.pone.0207292 (PMC6241125; doi:10.1371/journal.pone.0207292)
Supplement: S2 Table — (DOCX) [file pone.0207292.s002.docx]

**S2: Number of programs using at least one eHealth tool by categories^a^**

|  | All Programs N=85 |
| --- | --- |
| **Remote Consultation, Monitoring and Care** |  |
| Monitoring health status parameters by providers | 28 |
| Communication between care provider/patient (Including ePrescription) | 25 |
| Monitoring/interaction at distance (e.g. by video) | 23 |
| On-line appointment scheduling | 22 |
| Registration health status parameters by patients | 21 |
| **Self-management** |  |
| Electronic reminders | 22 |
| Computerized self-management tools | 21 |
| On-line decision supports | 3 |
| **Healthcare management** |  |
| Databases with patients’ health data | 54 |
| ICT-based communication between care providers | 40 |
| Systems providing warning messages/information | 30 |
| eReferral systems | 28 |
| Electronic reminders | 23 |
| EHRs | 60 |
| **Health Data Analytics** |  |
| Computerized decision supports | 30 |
| On-line decision supports | 13 |

^a^ Multiple answers were allowed
