# Supplementary material for: eHealth for people with multimorbidity: Results from the ICARE4EU project and insights from the “10 e’s” by Gunther Eysenbach
Source: PLoS One. 2018 Nov 14;13(11):e0207292. doi: 10.1371/journal.pone.0207292 (PMC6241125; doi:10.1371/journal.pone.0207292)
Supplement: S3 Table — (DOCX) [file pone.0207292.s003.docx]

**S3: Number of programs using at least one eHealth tool by some specific aspects^a^**

|  | All Programs N=85 |
| --- | --- |
| **Access to EHRs allowed to^b^:** |  |
| Medical care providers | 41 |
| Patients | 7 |
| **Training on the use of eHealth tools provided to:** |  |
| Care providers | 44 |
| Patients/carers | 20 |
| **Programs assured:**  Privacy/confidentiality of health data | 59 |
| Security of data | 50 |
| **Provision of (general) incentives for^c^:** |  |
| Providers | 28 |
| Patients | 18 |
| **Evaluation type^c^** |  |
| Internal | 59 |
| External | 28 |
| **Evaluation concerning^c^:** |  |
| Process | 58 |
| Outcomes | 36 |
| Cost-effectiveness | 25 |
| **Innovation** |  |
| eHealth tools specifically developed for the program | 30 |
| Existing tools were used | 20 |
| Existing tools adapted | 13 |

^a^ Multiple answers were allowed.

^b^ n=71

^c^ n=84
