# Supplementary material for: eHealth for people with multimorbidity: Results from the ICARE4EU project and insights from the “10 e’s” by Gunther Eysenbach
Source: PLoS One. 2018 Nov 14;13(11):e0207292. doi: 10.1371/journal.pone.0207292 (PMC6241125; doi:10.1371/journal.pone.0207292)
Supplement: S4 Table — (DOCX) [file pone.0207292.s004.docx]

**S4: Programs using at least one eHealth tool and focusing older people 65+. Benefits and barriers (number of agree)**

**S4A: Benefits of using eHealth tools^a^**

| Programs focusing 65+ with at least 1 eHealth tool, N=31 | | | |
| --- | --- | --- | --- |
|  | **N** |  | **%** |
| Management of care | 30 |  | 97 |
| Integration of care | 30 |  | 97 |
| Quality of care | 28 |  | 90 |
| Cost-efficiency | 24 |  | 77 |
| Quality of life | 23 |  | 74 |

**^a^** Multiple answers were allowed.

**S4B: Barriers for using eHealth tools^a^**

| Programs focusing 65+ with at least 1 eHealth tool, N=29 | | | |
| --- | --- | --- | --- |
|  | **N** |  | **%** |
| Lack of skills among providers | 16 |  | 55 |
| Inadequate technical ICT support | 16 |  | 55 |
| Lack of skills among patients | 15 |  | 52 |
| Inadequate legislative framework | 15 |  | 52 |
| Compatibility between different eHealth tools | 14 |  | 48 |
| Inadequate ICT infrastructures | 14 |  | 48 |
| Inadequate funding | 14 |  | 48 |
| Uncertainty of cost-efficiency | 11 |  | 38 |
| Privacy/security issues | 9 |  | 31 |
| Resistance by care providers | 8 |  | 28 |
| Cultural resistance | 7 |  | 24 |
| Resistance by patients | 6 |  | 21 |

**^a^** Multiple answers were allowed.
