# Supplementary material for: eHealth for people with multimorbidity: Results from the ICARE4EU project and insights from the “10 e’s” by Gunther Eysenbach
Source: PLoS One. 2018 Nov 14;13(11):e0207292. doi: 10.1371/journal.pone.0207292 (PMC6241125; doi:10.1371/journal.pone.0207292)
Supplement: S1 Text — (DOCX) [file pone.0207292.s005.docx]

**S1: Survey questions used in the study**

**ICARE4EU: Innovating care for people with multiple chronic conditions in Europe**

**Survey questions used in the study (for the paper)**

| V2. In which Country is the program implemented?  Elderly (programs focusing on elderly 65+) | | (deleted from the minimal dataset as potentially identifying information, when in combination with basic characteristics of the projects)  0 No  1 Yes | |
| --- | --- | --- | --- |
| V5. What are the main objectives of the program? *Please tick all boxes that apply* (0 No; 1 Yes) | | S7. Improving care coordination  S10. Increasing multi-disciplinary collaboration  S13. Improving patient involvement  S19. Reducing hospital admissions | |
| V7. How can the program best be characterized? *For each category please tick the box that applies best* | | V7a. *Integration level*   1. It is a small scale (pilot) project 2. It is a well-established & comprehensive program 3. It is fully integrated in the regular healthcare and/or support system   V7c. *Implementation level*   1. Local 2. Regional 3. National 4. Local/regional as part of a national program 5. Inter-/Supra-national 6. National as part of an international program | |
| V11. Which aspects of care or support for multimorbidity patients are addressed by the program? *Please tick all boxes that apply* (0 No; 1 Yes) | | S1 Lifestyle and health behaviour  S3 Prevention/delay of deterioration  S6 Medical care  S7 Nursing care | |
| V20. Please indicate which organizations/units are involved in the program. *Please tick all boxes that apply*  (0 No; 1 Yes) | | S1. University hospital  S2. General hospital  S3. Primary care practice | |
| V21. Please indicate which care providers (disciplines) are involved in the program. P*lease tick all boxes that apply* (0 No; 1 Yes)  **Types of eHealth tools used in the program.** | S1. General practitioners  S2. Medical specialists  S7. District / community nurses  S8. Hospital nurses / specialized nurses | |  |
| V35r. Remote Consultation, Monitoring and Care. *Please tick all boxes that apply* (0 No; 1 Yes) | | S1. Monitoring of health status parameters by providers  S2. Exchange of information on treatment and care between care provider and patient (e.g. video visits, e-visits, including ePrescription)  S3. Tele-monitoring through video/telephone/sensors  S4. On-line appointment scheduling  S5. Registration by patients of health status parameters (e.g. body temperature, heart rate, blood pressure, respiratory rate) using remote sensor/mobile devices | |
| V36r. Self-management. *Please tick all boxes that apply* (0 No; 1 Yes) | | S1. Electronic reminders (e.g. for appointment/consultation)  S2. Computerized self-management tool (e.g. for behavioural change)  S3. On-line (interactive) decision support | |
| V37r. Healthcare management. *Please tick all boxes that apply*  (0 No; 1 Yes) | | S1. Registration database consisting of patient data  S2. Exchange of information concerning common patients on treatment and care between different care providers (e.g. video conferences)  S3. Electronic information/warning aimed at patient safety  S4. eReferral systems  S5. Electronic reminders (e.g. appointment scheduling, available examination results) | |
| V38r. Health Data Analytics. *Please tick all boxes that apply* (0 No; 1 Yes) | | S1. Computerized decision support tool (e.g. for medication treatment)  S2. On-line (interactive) decision support | |
| V39A. Are Electronic Health Records (EHRs) used within the program?  *(only one box)*  V39B. Who have access to the EPRs?  *Please tick all boxes that apply*  (0 No; 1 Yes) | | 1. Yes, EHRs are already in use 2. We are planning to introduce EHRs 3. No, EHRs are not used in the program *(proceed to question 40)*   S1. Only relevant medical care providers  S3. Patients | |
| V40. Is training to use eHealth tools provided to (representatives of) patients as part of the program? | | 1. No 2. Yes | |
| V41. Is training to use eHealth tools provided to care providers as part of the program? | | 1. No 2. Yes | |
| V42. Which of the following aspects are addressed by the program? *Please tick all boxes that apply* (0 No; 1 Yes) | | S1. Data security / risk management of the devices and/or systems of the eHealth service (e.g. by means of a backup copy of the records and information).  S2. Privacy/ confidentiality of the electronic storage of administrative and medical patient data (e.g. by means of e-signature / unique ID / Electronic identification of patients and care providers) | |
| V43. Concerning only the eHealth tools applied in the program, please indicate to what extent you agree with the following statements concerning benefits of using eHealth:   1. eHealth tools improved the quality of care provided in the program 2. eHealth tools improved the quality of life of patients enrolled in the program (e.g. by home monitoring) 3. eHealth tools improved the cost-efficiency of the program 4. eHealth tools improved the integration of care in the program 5. eHealth tools improved management of care in the program   V43a-e_Rec | | 1 2 3 4  strongly disagree     strongly agree  strongly disagree     strongly agree  strongly disagree     strongly agree  strongly disagree     strongly agree  strongly disagree     strongly agree  1. Disagree (codes 1 and 2)  2. Agree (codes 3 and 4) | |
| V44. Concerning only the eHealth tools applied in the program, please indicate to what extent you agree with the following statements concerning barriers for using eHealth: The use of eHealth tools in the program was hampered by:   1. An inadequate national eHealth legislative framework 2. Inadequate funding 3. Inadequate ICT infrastructures 4. Inadequate technical/ ICT support 5. A lack of skills in using eHealth among care providers 6. A lack of skills in using eHealth among patients 7. Cultural resistance 8. Resistance by care providers 9. Resistance by patients 10. Uncertainty about cost efficiency 11. Compatibility between different eHealth tools 12. Privacy/security issues   V44a-l_Rec | | 1 2 3 4  strongly disagree     strongly agree  strongly disagree     strongly agree  strongly disagree     strongly agree  strongly disagree     strongly agree  strongly disagree     strongly agree  strongly disagree     strongly agree  strongly disagree     strongly agree  strongly disagree     strongly agree  strongly disagree     strongly agree  strongly disagree     strongly agree  strongly disagree     strongly agree  strongly disagree     strongly agree  1. Disagree (codes 1 and 2)  2. Agree (codes 3 and 4) | |
| V45. Innovation. *Please tick all boxes that apply* (0 No; 1 Yes) | | S1. eHealth tools been specifically developed for the program  S2. Existing eHealth tool(s) have been adapted  S3. Existing eHealth tool(s) were used | |
| V53. Are there incentives for care providers related to the program (e.g. additional financial support, additional staff)? | | 1. No 2. Yes | |
| V54. Are there incentives for patients included in the program (e.g. free access to devices/ services, medication, increased reimbursement)? | | 1. No 2. Yes | |
| V56A. Has the program been evaluated?  *Please tick all boxes that apply*  (0 No; 1 Yes)  V56B. If yes, please indicate what is evaluated. *Please tick all boxes that apply* (0 No; 1 Yes)  ***Empty cells in minimal data set are:***   - *not applicable for V39bs1 and s2;* - *missing values in other cases.* | | S1. Internally  S2. By an external organization  S1. The process of the program is evaluated  S2. Outcomes have been studied:  S4. Cost-effectiveness of the program has been studied | |
